# An Uncommon Aflibercept Side Effect: Full Thickness Macular Hole Formation After Intravitreal Injections in Patients With Pre-existing Vitreomacular Traction

**DOI:** 10.7759/cureus.12872

**Published:** 2021-01-23

**Authors:** Athanasios Karamitsos, Konstantina N Sorkou, Joy Bhagey, Roxane J Hillier, Vasileios T Papastavrou

**Affiliations:** 1 2nd Department of Ophthalmology, Aristotle University of Thessaloniki, Thessaloniki, GRC; 2 The Newcastle Eye Department, Royal Victoria Infirmary, Newcastle, GBR

**Keywords:** aflibercept intravitreal injection, full thickness macular hole, neovascular age-related macular degeneration, pigment epithelial detachment, vitreo-macular traction

## Abstract

Aflibercept is an intravitreally injected anti-vascular endothelial growth factor, commonly used in patients with several retinal pathologies, including neovascular age-related macular degeneration. We report a case series of three patients under treatment with an aflibercept regime for neovascular age-related macular degeneration, who were referred to vitreoretinal service between 2015-2016. In all cases, pre-existing vitreomacular traction was detected with an optical coherence tomography scan. All of them developed full-thickness macular hole after aflibercept intravitreal injections. The combined cataract and macular hole surgery was successful, with improvement in visual acuity. We suggest that dynamic alterations of the size of the pigment epithelium detachment resulting from intravitreal injections might intensify the pre-existing pathological adhesion of the vitreous-retinal interface and subsequently cause the formation of a full-thickness macular hole. Therefore, all practitioners treating patients with aflibercept intravitreal injections and pre-existing vitreomacular traction should be aware of the possible macular hole formation.

## Introduction

Aflibercept is a recombinant fusion protein consisting of vascular endothelial growth factor (VEGF) - binding portions from the extracellular domains of human VEGF receptors one and two, which are fused to the fragment crystallizable (Fc) portion of the human immunoglobulin G1 (IgG1). A high volume of aflibercept intravitreal injections is performed for several retinal pathologies, including neovascular age-related macular degeneration. Adverse effects from aflibercept intravitreal injections, as per product information provided by manufacturers, do not include macular hole formation.

There have been reported only a few cases that associate intravitreal injections of anti-VEGF drugs with macular hole formation; only one case concerns aflibercept, without being mentioned whether pre-existing vitreomacular traction was present [1, 2]. This is the first report of full-thickness macular hole formation in patients with pre-existing vitreomacular traction.

## Case presentation

Three patients with neovascular age-related macular degeneration, treated between 2015-2016, were included in the study. Optical coherence tomography (OCT) scan, SPECTRALIS HRA + OCT/Heidelberg Engineering (Franklin, MA, USA), was used to record both the vitreomacular traction and the full-thickness macular hole. Written informed consent was obtained from all patients. The research was approved by the Hellenic Data Protection Authority (Approval Number: 1808/ΓΝ/ΕΞ/75-2/01-02-2017) and followed the tenets of the Declaration of Helsinki.

Case 1

**Figure 1:**
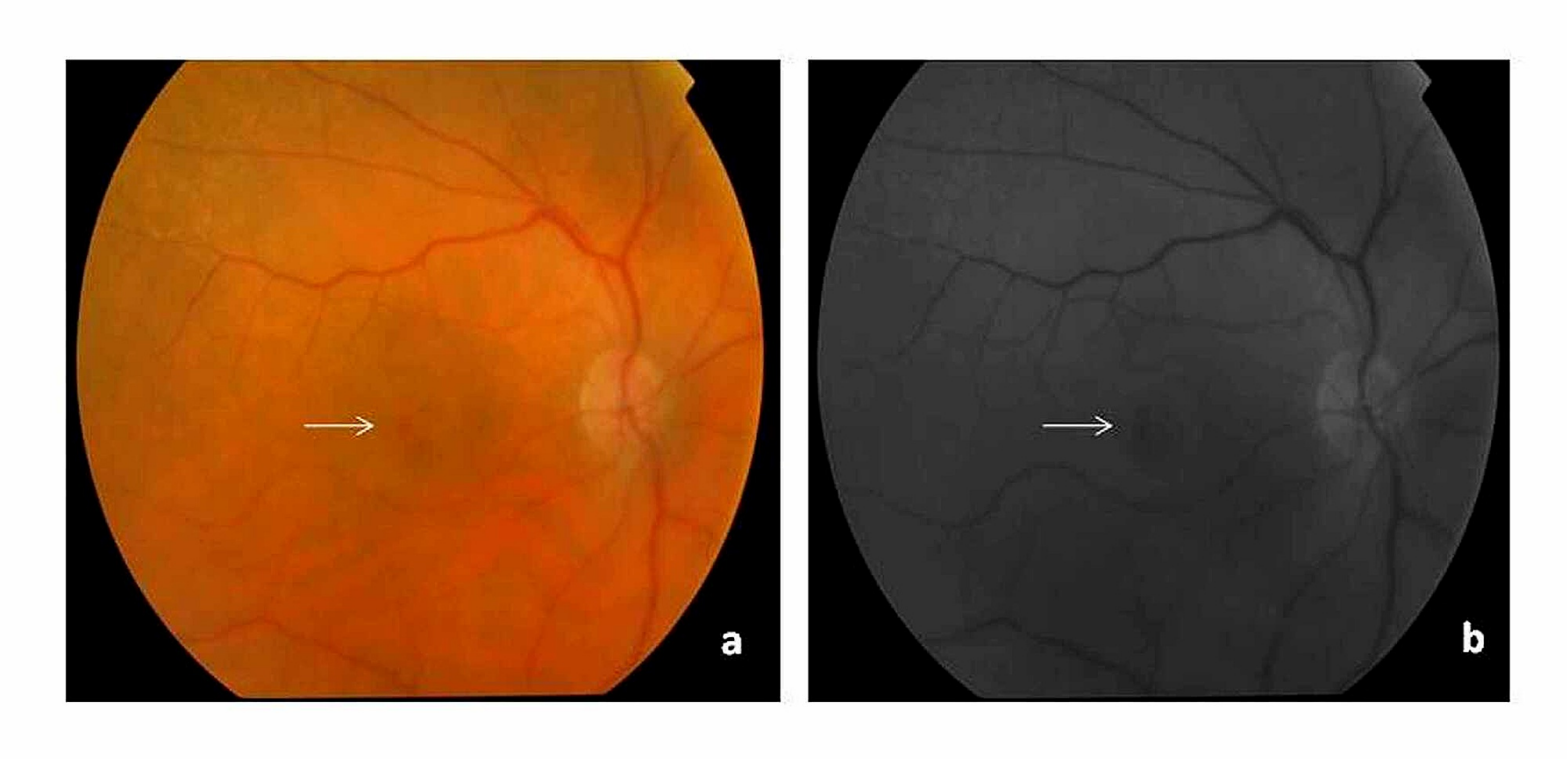
Fundus photographs from Case 1 a. Full-thickness macular hole after aflibercept intravitreal injection, indicated by arrow. b. Full-thickness macular hole after aflibercept intravitreal injection, indicated by arrow, with the use of red-free light.

A 65-year-old female, presenting best corrected visual acuity 6/24 in her left eye, was diagnosed with neovascular age-related macular degeneration and vitreomacular traction. The regime for neovascular age-related macular degeneration included monthly intravitreal injections of aflibercept in the left eye for four months. Two weeks after the second injection, a stage-three full-thickness macular hole was detected (Figure 1), with visual acuity decreased to 6/60. Combined cataract and macular hole surgery was successful and the vision in the left eye improved to 6/24.

Case 2

**Figure 2:**
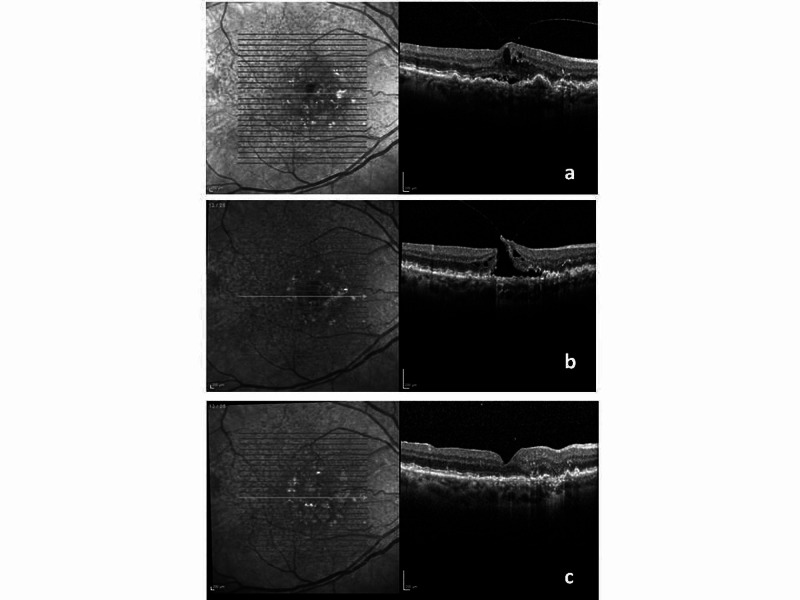
Optical coherence tomography (OCT) photographs from Case 2 a. Pre-injection OCT: Presence of subretinal fluid, intraretinal fluid, small pigment epithelial detachment, and also a small degree of vitreomacular traction. b. Post-injection OCT: Significant reduction of subretinal fluid, intraretinal fluid but formation of full-thickness macular hole. c. Post-cataract and macular hole surgery OCT: Complete closure of the full-thickness macular hole.

A 76-year-old female visited the emergency eye department with a one-week history of visual distortion in her right eye. The patient had an unremarkable past ocular history and her best corrected visual acuity was 6/18 in the right eye. OCT scan revealed the presence of subretinal fluid, intraretinal fluid, small pigment epithelial detachment, and also vitreomacular traction (Figure 2a). The diagnosis was neovascular age-related macular degeneration, and treatment with intravitreal injections of aflibercept was initiated. A week after the first injection, the patient attended the emergency eye department with symptoms of flashes and floaters, and her best corrected visual acuity dropped to 6/36 in the right eye. The patient underwent clinical examination, including examination of the peripheral retina. No evidence of peripheral retinal pathology was detected that potentially could justify the patient's symptoms. The patient underwent another OCT that revealed a significant reduction of the intraretinal fluid and the subretinal fluid, but full-thickness macular hole formation (Figure 2b). The patient referred to the vitreoretinal service and intravitreal aflibercept treatment resumed. Two months after the referral, the patient underwent combined cataract surgery and macular hole repair. Six weeks postoperatively, the best corrected visual acuity improved to 6/18 with complete closure of the full-thickness macular hole formation (Figure 2c).

Case 3

A 74-year-old male was treated with monthly aflibercept intravitreal injections for four months on account of neovascular age-related macular degeneration in the right eye. Vitreomacular traction was observed at initial diagnosis and the best corrected visual acuity was 6/36 preoperatively. One week after the second injection, a stage-three full-thickness macular hole was diagnosed with best corrected visual acuity being reduced to 6/60. Combined cataract and macular hole surgery was successful. Consequently, the vision in the right eye was increased to 6/36.

## Discussion

The posterior hyaloid interface, consisting of the posterior hyaloid membrane and the internal limiting membrane, has been implicated in a number of macular diseases. The posterior hyaloid membrane, which is a true basement membrane, consists of collagen IV. The internal limiting membrane consists of collagen types I and IV, proteoglycans, fibronectin, and laminin. It varies in thickness and composition. Firm vitreoretinal attachments are observed in areas with a thin internal limiting membrane as present at the vitreous base, optic disk, fovea, and over the major retinal blood vessels [3, 4].

The mechanism of idiopathic full-thickness macular hole, as proposed by Gass, includes focal tangential traction on the fovea, resulting from contraction of the pre-foveal vitreous cortex [5]. An OCT study of macular holes in 1999 by Gaudric et al. indicated that vitreous traction is possible oblique and therefore both tangential and anterior-posterior trans-vitreal traction have been implicated in idiopathic full-thickness macular hole development [6].

Researchers studying full-thickness macular hole formation after ranibizumab intravitreal injections in neovascular age-related macular degeneration have reported five cases with pre-existing pigment epithelial detachment and vitreomacular traction that treated with Lucentis®. In cases where fully thickness macular holes developed, there was no pattern as to when the hole developed after intravitreal injections began [1].

Querques et al. proposed that vitreoretinal traction may be a possible result of vitreous incarceration in the injection’s entry site, induced by intravitreal injection. Full-thickness macular hole formation may be caused by forces imposed on the retinal pigment epithelium and the retinal surface, due to contraction of the choroidal neovascular membrane [7].

Clemens et al. suggested that the structure of the vitreous gel is modified when chemical compounds are applied to the vitreous cavity. As a consequence, incomplete posterior vitreous detachment and vitreomacular traction are possible to appear, leading to macular hole formation [8].

Another hypothesis by Grigoropoulos et al. was that intravitreal injections probably increase the traction on the fovea by causing vitreous syneresis, globe deformation during needle insertion, and vitreous incarceration at the insertion spot. Focal sites of traction on the retinal surface may be created by incomplete posterior vitreous detachment induced during the intravitreal injection. In pre-existing vitreomacular traction, this may lead to a full-thickness macular hole [9].

Oshima et al. described a case of full-thickness macular hole development after three aflibercept intravitreal injections in a patient with neovascular age-related macular degeneration. In this specific case, a preexisting retinal pigment epithelium tear was detected, but no information about pre-existing vitreomacular traction is provided. The researchers suggested that the rolled retinal pigment epithelium flap associated with the subretinal fibrosis caused traction on the fovea during aflibercept treatment. This traction on the site of the fovea resulted in progression to a full-thickness macular hole [2].

In all three cases of the present study, pre-existing vitreomacular traction was detected in OCT. It is suggested that this pathological element may be a significant risk factor related to the pathophysiological mechanism of full-thickness macular hole formation. It is possible that elevation of the foveal retina and stretching of the photoreceptor layer by physical forces may occur due to subfoveal pigment epithelial detachment. As a result, the structural support of the fovea may be affected. This makes it more susceptible to vitreofoveal tractional forces, which can initiate a neurosensory retinal detachment and ultimately a full-thickness macular hole. Dynamic alterations of flattening and elevation of pigment epithelium detachment resulting from injections might intensify the pre-existing pathological adhesion of the vitreous-retinal interface and subsequently cause the formation of a full-thickness macular hole. Thus, it is proposed that full-thickness macular hole formation is more a mechanical phenomenon rather than an aftereffect of the anti-VEGF chemical structure itself.

This is the first report of full-thickness macular hole formation after aflibercept intravitreal injection in patients with neovascular age-related macular degeneration and pre-existing vitreomacular traction. All patients underwent successful macular hole surgery. Moreover, visual acuity improved significantly and stabilized after macular hole repair surgery in all patients. 

## Conclusions

Overall, full-thickness macular hole seems to be a potential complication of anti-VEGF intravitreal injections, aflibercept in our cases, in patients with vitreomacular traction, and this should be taken into consideration. Consequently, patients with age-related macular degeneration and pre-existing vitreomacular traction before initiation of treatment with anti-VEGF should be informed by their clinician about the risk of full-thickness macular hole formation.
